# Supplementary material for: Bifunctional Tm3+,Yb3+:GdVO4@SiO2 Core-Shell Nanoparticles in HeLa Cells: Upconversion Luminescence Nanothermometry in the First Biological Window and Biolabelling in the Visible
Source: Nanomaterials (Basel). 2020 May 21;10(5):993. doi: 10.3390/nano10050993 (PMC7279551; doi:10.3390/nano10050993)
Supplement: Supplementary file 1 [file nanomaterials-10-00993-s001.pdf]

nanomaterials

MDPI

Supplementary Information

Bifunctional Tm^{3+},Yb^{3+}:$GdVO_4$@SiO_2 Core-Shell Nanoparticles in HeLa Cells: Upconversion Luminescence Nanothermometry in the First Biological Window and Biolabelling in the Visible

Oleksandr Savchuk [1], Joan Josep Carvajal Marti [1,*], Concepción Cascales [2], Patricia Haro-Gonzalez [3], Francisco Sanz-Rodríguez [3,4], Magdalena Aguilo [1] and Francesc Diaz [1]

1 Fisica i Cristalografia de Materials i Nanomaterials (FiCMA-FiCNA)−EMaS, Universitat Rovira i Virgili (URV), Campus Sescelades, Marcelli Domingo 1, Tarragona E-43007, Spain; oleksandr.savchuk@urv.cat (O.S.); magdalena.aguilo@urv.cat (M.A.); f.diaz@urv.cat (F.D.)

2 Instituto de Ciencia de Materiales de Madrid, Calle Sor Juana Ines de la Cruz, Cantoblanco, 28049 Madrid, Spain; ccascales@icmm.csic.es

3 Fluorescence Imaging Group, Departamento de Fisica de Materiales, Facultad de Ciencias, Universidad Autónoma de Madrid, Madrid 28049, Spain; patricia.haro@uam.es (P.H.-G.); francisco.sanz@uam.es (F.S.-R.)

4 Departamento de Biología, Facultad de Ciencias, Campus de Cantoblanco, Universidad Autónoma de Madrid, Madrid 28049, Spain

* Correspondence: joanjosep.carvajal@urv.cat

Received: 23 April 2020; Accepted:17 May 2020; Published: 21 May 2020

Tm,Yb:$GdVO_4$@SiO_2 core-shell nanoparticles inside and outside HeLa cells.

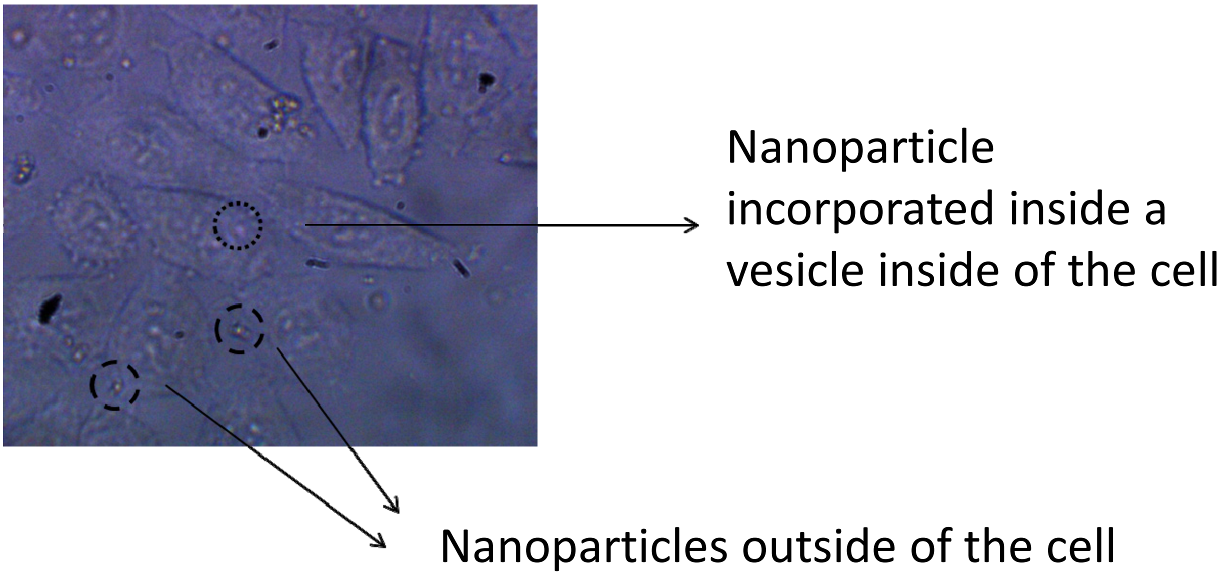

Figure S1. Microscope optical transmission image of the HeLa cells incubated with Tm,Yb:$GdVO_4$@SiO_2 core-shell nanoparticles when the excitation laser is on. The different arrows indicate the position were the core-shell nanoparticles are located. The nanoparticles located outside of the cell show a higher emission intensity, and they can be clearly seen in the images. The nanoparticles incorporated inside vesicles in the cells show a smaller emission intensity and a more attenuated image.

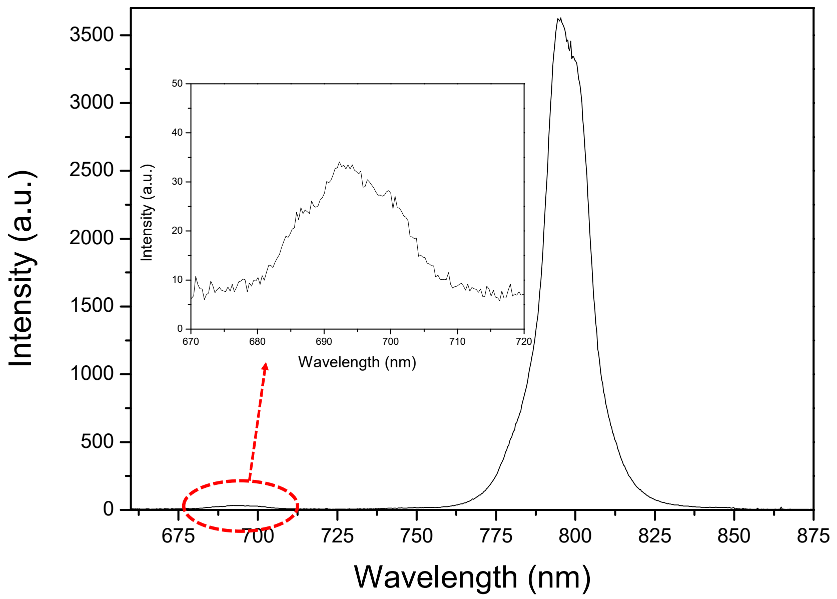

Figure S2. Typical luminescence spectrum recorded for the Tm,Yb:$GdVO_4$@SiO_2 core-shell nanoparticles in the I-BW after excitation at 980 nm with a power of 50 mW, and a spot size of 3 mm in the sample, to avoid photo damages in the living cells. From this spectrum the temperature could be determined by applying Equation (1) in the paper and the fitting function extracted from Figure 3a, resulting in a value of 308 ± 2 K after five measurements.

© 2020 by the authors. Submitted for possible open access publication under the terms and conditions of the Creative Commons Attribution (CC BY) license (http://creativecommons.org/licenses/by/4.0/).
